# Therapeutic Potential of Silver Nanoparticles (AgNPs) as an Antimycobacterial Agent: A Comprehensive Review

**DOI:** 10.3390/antibiotics13111106

**Published:** 2024-11-20

**Authors:** Nilakshi Barua, Alak Kumar Buragohain

**Affiliations:** 1 Department of Molecular Biology and Biotechnology, Tezpur University, Tezpur 784028, India; 2 Department of Microbiology, Faculty of Medicine, The Chinese University of Hong Kong, Prince of Wales Hospital, Shatin 999077, Hong Kong; 3 Department of Biotechnology, Royal Global University, Guwahati 781035, India

**Keywords:** nanoparticles, silver nanoparticles, antimycobacterial, tuberculosis, *Mycobacterium tuberculosis*, novel TB-therapy

## Abstract

The uncontrolled emergence of multidrug-resistant mycobacterial strains presents as the primary determinant of the present crisis in antimycobacterial therapeutics and underscores tuberculosis (TB) as a daunting global health concern. There is an urgent requirement for drug development for the treatment of TB. Numerous novel molecules are presently undergoing clinical investigation as part of TB drug development. However, the complex cell wall and the lifecycle of *M. tuberculosis* within the host pose a significant challenge to the development of new drugs and, therefore, led to a shift in research focus towards alternative antibacterial compounds, notably nanotechnology. A novel approach to TB therapy utilizing silver nanoparticles (AgNPs) holds the potential to address the medical limitations imposed by drug resistance commonly associated with currently available antibiotics. Their broad-spectrum antimicrobial activity presents the utilization of AgNPs as a promising avenue for the development of therapeutics targeting mycobacterial-induced diseases, which can effectively target *Mycobacterium tuberculosis*, including drug-resistant strains. AgNPs can enhance the effectiveness of traditional antibiotics, potentially leading to better treatment outcomes and a shorter duration of therapy. However, the successful implementation of this complementary strategy is contingent upon addressing several pivotal therapeutic challenges, including suboptimal delivery, variability in intra-macrophagic antimycobacterial effect, and potential toxicity. Future perspectives may involve developing targeted delivery systems that maximize therapeutic effects and minimize side effects, as well as exploring combinations with existing TB medications to enhance treatment outcomes. We have attempted to provide a comprehensive overview of the antimycobacterial activity of AgNPs, and critically analyze the advantages and limitations of employing silver nanoparticles in the treatment of TB.

## 1. Introduction

*Mycobacterium tuberculosis* (MTB) is the causative agent of tuberculosis (TB) and has infected approximately 25% of the human population globally [1]. TB is a significant global health threat and the leading infectious disease-related cause of mortality, surpassing HIV/AIDS. As one of the top ten causes of death globally, it requires urgent attention and action [2]. The World Health Organization (WHO) has added rifampicin-resistant *Mycobacterium tuberculosis* to the Critical priority list in 2024 [3]. The rise of multidrug-resistant *Mycobacterium tuberculosis* (MDR-MTB) strains and extensively drug-resistant tuberculosis (XDR-MTB), and the detrimental side effects of antituberculosis drugs have reignited interest in novel alternatives, such as nanoparticles (NPs) and natural compounds, as potential sources of novel antitubercular leads. In some cases, the mechanism behind the antimycobacterial activity of NPs has also been identified. Studies have shown that secondary metabolites from plants and other natural sources can inhibit the growth of MTB. However, the process of the isolation of active compounds has its drawbacks, like being time-consuming, the difficulty in cultivating the natural sources like plants, a low abundance, and complex chemical synthesis processes, hampering the competent production of valuable compounds [4,5,6]. The emerging fields of nanotechnology and nanoparticle science are bridging interdisciplinary fields of study, including chemistry, physics, and medicine, offering fresh perspectives and novel practical solutions for a number of pressing problems like bacterial infectious diseases [7].

**Figure 1:**
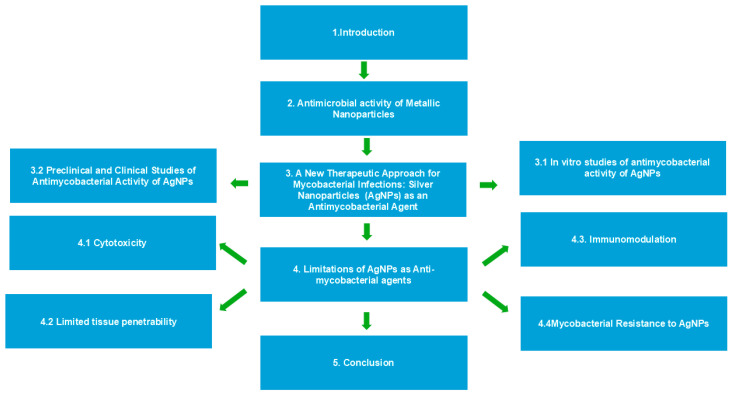
Overview of the review structure on silver nanoparticles (AgNPs) as antimycobacterial agents.

Nanotechnology is one of the most active study areas in contemporary material science and technology and includes several different types of nanostructured materials, including nanoparticles (NPs) [7], nanotubes [8], nanocomposites [9], and nanoconjugates [10]. In this comprehensive review, we will critically assess the current and prospective roles of silver nanoparticles (AgNPs) as a therapeutic agent against TB. AgNPs have gained recognition as effective agents against MTB due to their distinct antimicrobial properties. The mechanisms through which AgNPs exert their effects include the generation of reactive oxygen species (ROS) [11], disruption of cell membranes [12], interference with DNA [11] and protein synthesis [13], and the potential to enhance the efficacy of existing antibiotics. The diverse actions of AgNPs not only directly target the bacteria but also augment the effectiveness of conventional treatments, positioning them as a valuable alternative in addressing drug-resistant strains of MTB. AgNPs induce oxidative stress in bacterial cells by generating ROS, which can damage cellular components such as lipids, proteins, and DNA, ultimately leading to cell death. The oxidative stress induced by ROS disrupts the redox balance within bacterial cells, thereby amplifying the bactericidal efficacy of silver nanoparticles (AgNPs) [11,12,13]. AgNPs interact with bacterial cell membranes, causing structural damage and increasing permeability. This results in the leakage of cellular contents and ultimately leads to cell lysis [11,12]. The size and shape of nanoparticles influence their interaction with cell membranes, as smaller particles possess a greater surface area for engaging with bacterial cells [14]. AgNPs can bind to bacterial DNA, leading to structural changes that inhibit replication and transcription processes [11]. They disrupt protein synthesis by binding to ribosomal subunits, which interferes with the translation process and causes protein dysfunction [13]. AgNPs have been shown to restore the effectiveness of antibiotics, such as rifampicin, against resistant strains of MTB. They achieve this by increasing the permeability of the bacterial cell wall, which allows for a better penetration of the antibiotic. Combining AgNPs with antibiotics can reduce the necessary dosage of these medications, potentially minimizing side effects and slowing the development of antibiotic resistance [15]. Our review will probe into the various mechanisms of silver nanoparticles that make them a promising candidate for treating this infectious disease and examine the potential challenges and limitations that must be overcome for successful clinical implementation. Figure 1 provides an overview of the review structure.

## 2. Antimicrobial Activity of Metallic Nanoparticles

The increased resistance of microbes to current antimicrobial treatments has resulted in a rise in multidrug-resistant infections and contributes to significant morbidity across all age groups of patients [16]. Metallic nanoparticles have potential prospects for use in treating infectious diseases due to their distinctive antiviral, antibacterial, and antifungal characteristics [17]. There are several classes of nanoparticles that demonstrate intrinsic antibacterial and antibiofilm properties [18], including metallic nanoparticles such as copper [14,19], iron [20], gold [21], zinc [22] or silver-based ones [23], carbon nanotubes [24], polycationic polymers, and metallic nanoparticles, such as chitosan-gold NP [25,26].

Recently, antibacterial peptides and nanoparticles have received particular attention in the search for more effective antimycobacterial agents [27]. Silver nanoparticles (AgNPs) have received the most attention among antibacterial nanoparticles because of their potent antibacterial action, and unlike antibiotics, most pathogenic bacteria currently known rarely develop resistance to metallic nanoparticles [28]. However, in some cases, resistance to metallic nanoparticles has been noted and will be covered in a separate section.

AgNPs have exhibited broad-spectrum and high antimicrobial activity, killing many pathogens even at very low concentrations [22], including (i) bacteria such as *Escherichia coli, Klebsiella pneumonia*, and *Staphylococcus aureus*; (ii) fungi such as *Candida albicans* and *Aspergillus niger*; and (iii) viruses such as Hepatitis B virus (HBV) and HIV. AgNPs are the most frequently used anti-infectious nanoparticle adjuvants among metallic nanoparticles [29] or chitosan [30]. AgNPs have also shown potent activity against methicillin-resistant *S. aureus* (MRSA) [31]. AgNPs synthesized from the silk sericin (SS) protein, which is produced by the moth *Bombyx mori*, showed antibacterial activity against *E. coli* and *P. aeruginosa* at minimal inhibitory concentrations of 20 μg/mL [32]

AgNPs of pure crystalline nature produced by the metallotolerant fungal strain, *Penicillium notatum* (K1) exhibited excellent antibacterial activity against both Gram-positive and Gram-negative bacteria, including *Bacillus subtilis*, *S. aureus* (ATCC9144), *Listeria innocua* (ATCC13932), *E. coli* (ATCC10536), *P. aeruginosa* (ATCC10145), and *Enterococcus faecalis* [33] AgNPs produced using extracts and supernatants of *Trichoderma harzianum* and *Ganoderma sessile,* respectively, exhibited in vitro antibacterial effects against *E. coli*, *P. aeruginosa,* and *S. aureus* with a MIC varying from 1.26 to 5.0 µg/mL [34]

## 3. A New Therapeutic Approach for Mycobacterial Infections: Silver Nanoparticles (AgNPs) as an Antimycobacterial Agent

Nanotechnology presents a novel antimycobacterial therapeutic approach which involves enhancing the efficacy of the antibiotics already in use by developing new formulations, such as liposomes, niosomes, dendrimers, hydrogel nanoparticles, and solid lipid nanoparticles. These novel formulations aim to improve the therapeutic attributes of both first- and second-line antibiotics, facilitating better targeting of the pathogens and more effective drug release. By employing these sophisticated formulations, the potency of antimycobacterial medications can be boosted but also improve overall treatment outcomes, leading to more successful therapies for challenging mycobacterial diseases [35]. In the following two sections we aim to discuss the outcomes of the in vitro, preclinical, and clinical studies conducted to evaluate the antimycobacterial activity of AgNPs.

### 3.1. In Vitro Studies of Antimycobacterial Activity of AgNPs

Recent in vitro experiments [36,37,38,39,40,41,42,43,44,45,46,47,48,49,50,51,52,53,54,55,56,57,58,59,60,61,62,63] have provided evidence that AgNPs possess antimycobacterial activity (Table 1). The use of AgNPs to enhance anti-TB drugs shows promise in combating drug-resistant strains of MTB. AgNPs have demonstrated the potential to improve the effectiveness of current anti-TB medications, especially in cases of developed resistance. This method utilizes the distinct properties of nanoparticles to improve drug delivery and efficacy, providing a new approach to tackle the issues associated with multidrug-resistant TB. The use of AgNPs in combination with existing anti-TB drugs like rifampicin and moxifloxacin has been demonstrated to enhance the efficacy of these drugs against resistant strains. For instance, a composite of rifampicin and AgNPs completely inhibited the growth of rifampicin-resistant strains in 51.4% of cases, with higher concentrations achieving a 100% bactericidal effect. AgNPs, when used in conjunction with traditional TB medications like fluoroquinolones (FQs), particularly moxifloxacin (Mfx), significantly enhanced the antibacterial efficacy against 50 resistant strains of MBT as determined by the BACTEC™ MGIT 960™ TB System. Adding Mfx and silver nanoparticles to FQ-resistant MTB strains led to a complete suppression of MTB growth in 54.3% of cases. When a 20 nm AgNP 2.5% suspension was used along with a standard dose of Mfx, it resulted in a bactericidal effect in 90% of cases, and 100% effectiveness was achieved when 5% and 10% suspensions were utilized [36]. Combining rifampicin and silver nanoparticles resulted in a complete suppression of growth in rifampicin-resistant MTB strains in 51.4% of cases, with higher concentrations showing a bactericidal effect in all the strains tested [37]. A study evaluated the effectiveness of silver nanoparticles (AgNPs) against MTB H37Rv and *M. bovis*, including a multiple drug-resistant (MDR) strain and a few selected clinical isolates. The study determined the minimum inhibitory concentration (MIC) of the AgNPs through microplate Alamar blue assay. The results indicated that the synthesized AgNPs effectively inhibited the proliferation of all tested strains, including two reference strains and one MDR strain. The MIC for the MDR strain ranged from 2 to 12 µg/mL, while the MIC for MTB H37Rv and *M. bovis* ranged from 2 to 14 µg/mL and 3 to 30 µg/mL, respectively [38]. Biogenic AgNPs synthesized using a leaf broth of *Limonia acidissima* L. inhibited MTB at a low MIC (3.12 µg/mL), highlighting the potential of AgNPs for developing novel TB treatments [39]. AgNPs synthesized by the enzymatic reduction of silver nitrate (AgNO_3_) solution exhibited potent antimycobacterial activity against clinical isolates of MDR-MTB, XDR-MTB, and *Mycobacteria* Other Than Tuberculosis (MOTT) strains within the MIC range of 6.25 to 12.5 μg/mL determined through the Microplate Alamar Blue Assay (MABA). Alginate-capped AgNPs demonstrated significant anti-mycobacterial activity in the THP-1 macrophage infection model against cytosolic MTB and effectively inhibited the growth of dormant-like MTB in vitro [40]. Two mesoporous silica nanoparticles containing silver bromide nanoparticles (MSNs-AgBrNPs) demonstrated antimycobacterial activity against the MTB H37Rv strain [41]. Song et al., in 2006, evaluated non-biogenic AgNPs measuring less than 10 nm against various bacteria species, including MTB, *E. coli*, *S. aureus*, and *Salmonella typhi*. The antimycobacterial impact was observed at 10 ppm, and the postulated mechanism was that AgNPs penetrated the cytoplasm and led to subsequent metabolic abnormalities of the bacteria [42]. A study recently revealed that phytogenic AgNPs had higher activity against mycobacteria and low cytotoxicity (10 times the dose indicated as the MIC for MTB) on infected macrophages [43]. Studies using biogenic AgNPs synthesized using plants showed potent in vitro antimycobacterial effects by inducing 99% cell death in four *Mycobacterium* species tested, namely, MTB (MTCC-300), *M. pheil* (MTCC-1723), *M. avium* (MTCC-1724), and *M. smegmatis* (MTCC-994) [44]. Colloidal silver nanoparticles synthesized by one-step green reduction at room temperature using *Catharanthus roseus* exhibited bactericidal activity with inhibition zones of 24 ± 0.04 mm and 22 ± 0.62 mm against *M. smegmatis* and MTB, respectively [45]. In vitro, the evaluation of silver nanoparticles synthesized from the fruits of *Coriandrum sativum* by Microplate Alamar Blue Assay (MABA) showed remarkable antitubercular activity with the inhibition of MTB H73Rv at a concentration of 1.6 μg/mL [46]. AgNPs synthesized by a bioreduction of silver nitrate (AgNO_3_) solution with *Cucumis sativus* exhibited antimycobacterial activity against MTB H37Rv and 26 clinical isolates, which included drug-sensitive (DS), MDR-MTB, XDR-MTB, and MOTT, by BACTEC 460TB radiometric analysis with a MIC range of 7.8 to 15.6 µg/mL and MOTT with a MIC of 25 µg/mL [47]. AgNPs synthesized using the leaf extract of *Ipomea carnea* exhibited potent activity against *M. smegmatis* with an inhibition zone of 14 mm [48]. AgNPs synthesized using the leaf extract of *Psidium guajava L*. exhibited antimycobacterial activity against MTB, *M. smegmatis,* and *M. phlei* with inhibition zones 13.73 ± 0.12 mm, 12.53 ± 0.42 mm, and 14.23 ± 0.69 mm respectively [49]. Spherical AgNPs biosynthesized by utilizing the alcoholic extract of *Plumbago auriculata* showed antitubercular activity against MTB with a MIC value of 1.6 μg/mL [50]. AgNPs green synthesized by the bioreduction of AgNO_3_ with *Rhizopus stolonifera* enzymes exhibited anti-tubercular activity within the MIC range of 6.25 to 12.5 μg/mL against MDR-MTB, XDR-MTB, and MOTT strains when determined using the Microplate Alamar Blue Assay (MABA) [51].

In contrast, physiochemically synthesized AgNPs were reported to inhibit MDR and XDR strains of MTB in vitro at dosages as low as 1 µg/mL [52]. The co-administration of rifampicin with AgNPs resulted in a 68% reduction in MTB colony-forming units in intracellular mycobacteria. However, although AgNPs internalized within the phagolysosomal machinery THP-1 macrophages, they exhibited low antitubercular effects for the intracellular mycobacteria in the absence of rifampicin [53]. Spherical AgNPs measuring 13 nm failed to exhibit an antibacterial effect against intracellular MTB internalized by macrophages. However, in combination with Zn (5_Ag_:5_ZnO_), they showed an intracellular antibacterial activity. Additionally, 5_Ag_:5_ZnO_ showed no evident impairment to normal lung (MRC-5) cell lines [54]. Spherical, biogenic AgNPs synthesized with *Alstonia macrophylla* and *Trichoderma* sp., and coupled with antimicrobial peptides in levels of 0.1 and 0.5 ppm exhibited antitubercular action against intracellular *M. marinum* and *M. smegmatis* phagocytosed by macrophages. The enhanced antitubercular activity was not correlated with high NO levels, and it was postulated that the production of superoxide radicals and the activation of macrophages by cytokines played a role in antimycobacterial activity. It was also observed that upon combination with rifampin, AgNP antimycobacterial activity was enhanced when tested against *M. smegmatis* [55]. The green-synthesized silver nanoparticles using *Clerodendrum serratum* extract, especially when encapsulated with polyethylene glycol (PEG), exhibit promising anti-mycobacterial and anti-biofilm activities against *M. smegmatis*, *M. fortuitum,* and *M. marinum* [56]. AgCl-NPs synthesized using commercial yeast extract exhibited promising antimycobacterial activity against MTB H37Rv and *M. smegmatis* MC2155 at 37 µg/mL. AgCl-NPs at the concentration of 37 µg/mL inhibited 92% of MTB H37Rv growth compared to a 95% growth reduction by rifampicin at 2 µg/mL [57]. Biogenic spherical AgNPs with the size of 20–56 nm, which were synthesized using *Sesbania grandiflora,* demonstrated activity against MTB H37Rv at 12.5 µg/mL [58]. Green synthesized AgNPs using *Citrus pseudolimon* fruit peel extract inhibited the growth of MTB. When treated with AgNPs, these bacteria exhibited growth only at the initial time point (0 h), and no viable cells were detected after 6 h of treatment, indicating potent anti-mycobacterial activity [56,59]. AgNPs synthesized from *Syzygium aromaticum* seeds inhibited the growth of *Mycobacterium smegmatis* at an MIC of 250 μg/mL [60]. Spherical chitosan-coated AgNPs killed intracellular *M. smegmatis* internalized in RAW264 at a concentration of 3 ppm, (in both pre- and post-exposure treatments) and exhibited most of the antimycobacterial effect within the first hour of incubation. The inhibition of growth was observed to be concentration-dependent as well as time-dependent, indicating that the antimycobacterial activity was mediated via cell membrane disruption. Additionally, chitosan-coated AgNPs did not exhibit cytotoxicity against the RAW264.7 cell line at the bactericidal dose [61]. Combining AgNPs with peptides or chitosan for antimycobacterial activity is an intriguing tactic, as demonstrated by spherical N,N,N-trimethyl chitosan chloride (TMC)/AgNPs in a dose from 0.98 to 125 mg/mL via the disruption of the cell wall of MTB [62]. Additionally, it was demonstrated that spherical polyvinylpyrrolidone and bovine serum albumin (BSA)-capped AgNPs with sizes of 6–45 nm and 5–9 nm, respectively, have bactericidal effects on both clinically isolated and standard MTB strains. Mycobacterial cell membrane damage and subsequent bacterial lysis were the mediators of the antibacterial effect [63]. In vitro, the evaluation of the antibacterial activity of spherical AgNPs measuring 3–60 nm at the concentration of 5, 25, and 50 µg/mL exhibited potent activity against clinical strains of MDR-MTB [63]. AgNPs measuring 10–150 nm were observed to enhance the efficacy of the antibacterial drugs isoniazid, rifampicin, ethionamide, levofloxacin, ofloxacin, and kanamycin against 1164 clinical isolates of MTB in addition to the direct antitubercular activity in concentrations starting at 5 mg/L [64]. Chemically synthesized tetrahedral and spherical AgNPs measuring 50 nm showed antimycobacterial activity against standard and clinical isolates of MTB and *M. bovis* with a lower minimum inhibitory concentration (MIC) for standard cultures of MTB and *M. bovis* (1 and 4 µg/mL) and the MIC was comparatively higher in the clinical isolates, ranging from 1 to 16 µg/mL for MTB and 4 to 32 µg/mL for *M. bovis* [38]. The majority of the investigated AgNPs exhibit concomitant antimycobacterial activity against several species, including M. TB, *M. phlei*, *M. avium*, and *M. smegmatis*, despite reports that distinct mycobacterial species differ in their sensitivity to AgNPs [44].

The size and shape of the AgNPs have a significant impact on their antimycobacterial activity, influencing their interactions with bacterial cells. Smaller nanoparticles typically demonstrate enhanced antimicrobial efficacy due to their increased surface area-to-volume ratio, which facilitates improved interactions with and penetration into bacterial cells. Furthermore, the geometric shape of the nanoparticles is critical, as specific morphologies may offer superior antimicrobial properties. Consequently, these morphological characteristics are essential determinants of the effectiveness of AgNPs against Mycobacterium tuberculosis, the pathogen responsible for tuberculosis (TB). Smaller silver nanoparticles (AgNPs), particularly those with an average size of 9.49 nm, demonstrate significant antibacterial activity. This enhanced effectiveness is attributed to their improved capacity to penetrate bacterial cells and disrupt essential cellular functions [65]. In recent studies, silver nanoparticles (AgNPs) with a size of approximately 45 nm have demonstrated a significant ability to inhibit the growth of MTB strains, including those that are multidrug-resistant. The minimum inhibitory concentrations (MICs) observed for these nanoparticles ranged from 2 to 30 µg/mL, indicating their potential as an effective treatment option against resistant tuberculosis strains [66,67]. The shape of AgNPs influences their interaction with bacterial cells. Tetrahedral-shaped nanoparticles have shown effective antimycobacterial activity against MTB [66,67]. Nanoparticles with distinct geometries, such as rods and cubes, demonstrate enhanced efficacy at lower concentrations compared to their spherical counterparts. This increased effectiveness is likely attributable to their greater surface reactivity and enhanced capacity to disrupt bacterial membranes [68]. Silver nanoparticles (AgNPs) exhibit significant antimycobacterial properties by enhancing cell wall permeability, which ultimately leads to cellular damage and death. This mechanism has been documented in studies involving alginate-capped AgNPs, demonstrating their effectiveness against both drug-resistant and dormant strains of Mycobacterium tuberculosis [40]. The antimicrobial properties of silver nanoparticles (AgNPs) arise from their capacity to generate reactive oxygen species and interfere with cellular processes. This activity is amplified in nanoparticles of smaller sizes and certain shapes [69,70]. Smaller and uniquely shaped silver nanoparticles (AgNPs) exhibit promising efficacy against mycobacterial infections. However, it is essential to address the potential cytotoxicity associated with these nanoparticles. Studies suggest that the toxicity of AgNPs to human cells is influenced by their shape and size. Consequently, while AgNPs serve as effective antimicrobials, their design must strike a balance between effectiveness and safety to minimize any adverse effects [69].

In vitro studies have demonstrated that silver nanoparticles possess significant antimycobacterial properties, effectively inhibiting the growth of various bacterial strains. These findings indicate that silver nanoparticles could serve as a promising alternative for addressing antibiotic-resistant infections in clinical environments. Alginate-capped silver nanoparticles (AgNPs) and mesoporous silica nanoparticles with silver bromide are effective against Mycobacterium tuberculosis (MTB), including dormant forms. Plant extract-based AgNPs demonstrate significant antimicrobial properties and low cytotoxicity while targeting mycobacteria. Physiochemically synthesized silver nanoparticles (AgNPs) are effective against multidrug-resistant (MDR) and extensively drug-resistant (XDR) strains at low dosages. However, some spherical AgNPs lack antibacterial effects against intracellular Mycobacterium tuberculosis (MTB) without antibiotics. The enhancement of antimycobacterial activity relies on superoxide radicals and cytokine activation. Overall, AgNPs, especially those from natural sources, show promise as therapeutic agents against mycobacterial infections.

In vivo Preclinical and Clinical Studies.

### 3.2. Preclinical and Clinical Studies of Antimycobacterial Activity of AgNPs

Only a few in vivo investigations [40,70,71,72] (Table 2) have been conducted to date compared to the enormous number of in vitro studies investigating the potential use of AgNPs as antimycobacterial medicines. An in vivo study showed that inhaling silver nanoparticles with a size of 43.6 ±10.7 nm stabilized with PVP led to a noticeable bactericidal effect in the chronic tuberculosis C57B1/6 mice model. The nanoparticles suppressed mycobacterial growth in vitro by two times at a concentration of 50 μg/mL. Additionally, inhaling the silver nanoparticles reduced mycobacterial growth and inflammation in the lungs of C57B1/6 mice infected with tuberculosis. The silver nanoparticles also restored the balance of the immune system, increased the production of reactive oxygen species by neutrophils, and decreased the levels of inflammatory cytokines INF-γ, TNF-α, and IL-4 in the blood and lung fluid [37]. Alginate was used to synthesize AgNPs in a green and biocompatible way and was demonstrated to be effective against drug-resistant and dormant MTB H37Rv strains via increasing the cell-wall permeability. In vivo, tests on AB/TL wild-type zebrafish (*Danio rerio*) embryos and larvae and female BALB/c mice showed potential as a new therapeutic option for TB treatment. The addition of alginate enhanced the biocompatibility of AgNPs and eliminated the need for toxic chemicals [40]. Lung histology and survival rates in 68 inbred BALB/c murine infection models demonstrated that, in the rodent trial, the combination of isoniazid and silver nanoparticles was preferable compared to AgNPs used alone at the concentrations of 5, 25, and 50 µg/mL [61]. The effectiveness of AgNPs with sizes of 10–150 nm as single therapy molecules or in combination with isoniazid was examined in an in vivo study using 65 mice that had been experimentally infected with clinical MDR strains of MTB. The combined isoniazid and AgNP treatment resulted in a 95% survival rate for TB-infected animals and a 35% survival rate for the group receiving only AgNP, compared to a 100% death rate in the control group infected with MTB [64]. Another study by the same authors used three experimental groups of MDR-TB-infected mice to test the synergy of isoniazid combined with AgNPs. Group 1 received isoniazid (50 mg/kg), group 2 received AgNPs at doses ranging from 12.5 to 125 µg/kg intramuscularly, and group 3 received a combination of the treatments described for groups 1 and 2. In group 3, isoniazid was administered at a dose of 50 mg/kg concurrently with nanoparticles at the doses previously specified in group 1 and group 2.

Based on the histopathologic grading of lesions, when treating MDR-TB strains, using AgNPs improved the effectiveness of isoniazid [70].

A clinical study carried out at the Karachaevo-Cherkessian TB dispensary with 50 human subjects evaluated the effectiveness of nano-scale silver preparations for treating tuberculosis. Two different preparations, argovit-C and vitargol, were tested for their bactericidal activity against drug-resistant mycobacteria at various concentrations of isoniazid. It was found that the 3.3% solution of argovit-C had 100% bactericidal activity and was therefore chosen for the clinical study. The study showed that the inhalation of the 3.3% argovit-C solution twice daily for 10 min over 2 months was more effective in treating laryngeal tuberculosis than standard anti-tuberculosis treatment. The findings of the study indicated that 93.3% of patients in the AgNPs group tested negative for MTB in sputum culture after two months of treatment, surpassing the 70% rate observed in patients receiving conventional anti-TB medication. Furthermore, patients in the AgNPs group exhibited an accelerated healing of laryngeal TB lesions, encompassing ulcerations, and improved voice function in comparison to those administered standard anti-tuberculosis medication [73]. The AgNPs demonstrated their potential in both pre-clinical settings in mice and zebrafish as well as in clinical settings, highlighting their ability to improve the effectiveness of existing treatments and provide new therapeutic options. However, there are significant limitations and challenges that must be addressed before these nanoparticles can be widely used in clinical practice. Further clinical studies are necessary to assess the effectiveness and potential applications of AgNPs in antimycobacterial therapy.

## 4. Limitations of AgNPs as Anti-Mycobacterial Agents

### 4.1. Cytotoxicity

AgNPs are widely utilized for their antimicrobial properties; however, concerns have been raised regarding their potential toxicity to eukaryotic cells. The majority of inorganic nanoparticles, including AgNPs, have the potential to be toxic, which may restrict their use in biological contexts [74]. However, despite the expansion of use in recent decades, the evidence for AgNP toxicity is still not apparent [75]. Studies indicate that AgNPs can induce cytotoxicity, oxidative stress, DNA damage, and other adverse effects in various eukaryotic systems. A potential mechanism of toxicity is suggested, involving the disruption of the mitochondrial respiratory chain by AgNP, leading to the production of ROS and the interruption of ATP synthesis, which in turn causes DNA damage [76].

If the cellular protective antioxidative mechanisms are compromised, the increased production of ROS, which is one of the antibacterial mechanisms of AgNPs, can be detrimental to normal cells and cause inflammation, autophagia, apoptosis, necrosis, irreversible DNA damage, mutations, and perhaps oncogenesis [73]. AgNPs have exhibited cytotoxicity in human dermal fibroblasts and exert an influence on cellular processes by modulating microRNA expression, thereby disrupting cytoskeleton integrity, reducing ATP synthesis, and triggering apoptosis [77]. AgNPs have been shown to induce DNA strand breaks and oxidative DNA damage in various human cell lines, including hepatoma, leukemia, and fibroblasts. A size-dependent effect has been observed where smaller nanoparticles (4.7 nm) exhibit more genotoxicity than larger ones (42 nm). Silver nanoparticles (AgNPs) have demonstrated a dose-dependent cytotoxic effect on Chinese hamster ovary (CHO) cells, with an observed IC50 of 68.0 ± 2.65 μg/mL following a 24 h exposure period. This cytotoxicity has been associated with oxidative stress, as evidenced by heightened levels of reactive oxygen species (ROS) and compromised mitochondrial function [78,79]. A high antibacterial activity and low toxicity for the investigated doses have also been observed, e.g., AgNPs synthesized from *Phenerochaete chrysosporium* exhibited high antibacterial activity against *P. aeruginosa*, *K. pneumoniae*, *S. aureus*, and *S. epidermidis*, but did not exhibit cytotoxicity in mouse embryo fibroblasts at doses up to 12.5 µg/mL AgNPs [80].

AgNPs disrupt telomere dynamics in cancer cells and activate DNA damage response pathways, suggesting potential therapeutic applications but also highlighting their genotoxic potential. AgNPs have also exhibited neurotoxic effects in glutamatergic neurons derived from human embryonic stem cells. Citrate-coated AgNPs significantly increased ROS production and impaired neurite outgrowth, leading to neurodegenerative changes comparable to those observed in Alzheimer’s disease [81]. However, no cytotoxicity was observed against macrophages in mycobactericidal concentrations of (0.1 and 0.5 ppm) of biogenic AgNPs generated from *Alstonia macrophylla* and *Trichoderma* sp. Additionally, exposure to larger doses of AgNPs resulted in cytotoxicity and DNA damage [55]. The AgNPs produced using extracts and supernatants of *Trichoderma harzianum* and *Ganoderma sessile* exhibited antibacterial effects against *E. coli*, *P. aeruginosa,* and *S. aureus*. The MIC ranged from 1.26 to 5.0 µg/mL, varying with the bacterial strain and the type of nanoparticle utilized. Upon exposure for 24 h to varying concentrations of AgNPs, fibroblast viability remained above 50%, even when exposed to AgNPs concentrations of up to 20 µg/mL. Conversely, AgNPs demonstrated heightened cytotoxicity against macrophages, leading to decreased viability at concentrations of 10 µg/mL [34]. AgNPs induced inflammatory responses in monocytes and keratinocytes, exhibiting varying effects depending on cell type. They also promote caspase activity and necrosis, indicating potential immunotoxicity [82]. The evaluation of the toxicity of AgNPs in the nematode *C. elegans* revealed a significant reduction in both the number of live nematodes and their body length [83]. While AgNPs show potential for various applications, their potential toxicity to eukaryotic cells requires careful consideration. The variability in effects across different cell types and environments emphasizes the need for further research to fully understand and mitigate these risks.

### 4.2. Limited Tissue Penetrability

The limited permeability of AgNPs in tuberculous granulomas has a significant impact on treatment outcomes. This is because it affects the efficiency of drug delivery and therapeutic efficacy. Granulomas are complex structures that form in response to MTB, and they create a barrier to effective drug penetration. This challenge is made even more difficult by the unique properties of silver nanoparticles. While they show promise in their antimicrobial capabilities, they face limitations in penetrating these dense structures [84]. Considering the inadequate vascularization of tuberculose lung cavities found in chronic tuberculosis cases, a low intra-lesional accumulation is also anticipated in the absence of sufficient functionalization following an intravenous delivery route [85]. Additionally, the ability of mycobacteria to survive in an environment where non-vascularized necrotic material is largely inaccessible to large molecules and immune system cells within the granuloma core can ensure that a reservoir of live mycobacteria is present in a biological setting [86]. The permeability of AgNPs within granulomas, particularly in the context of TB, is influenced by a range of critical factors, including nanoparticle dimensions, surface properties, and the surrounding biological milieu. A study using the zebrafish model infected with *M. marinum* has shown that different nanoparticles, like PEGylated liposomes, can move from the blood vessels and target granulomas. Also, larger particles (~700 nm) tend to accumulate at the infection sites [87]. The efficacy of AgNPs in penetrating granulomas and their potential therapeutic application in the treatment of TB pose notable challenges. Although AgNPs exhibit promise in addressing drug resistance in mycobacterial infections, their consistency in delivery and intracellular effectiveness within macrophages varies, and concerns regarding potential residual toxicity persist.

The interaction between AgNPs and biological tissues is intricate. For instance, AgNPs have demonstrated varying inflammatory responses within the human gastrointestinal tract, dependent on the size of the nanoparticles. This observation suggests that biological diversity plays a pivotal role in influencing the behavior and efficacy of nanoparticles [88]. Despite their antimicrobial properties, the widespread use of AgNPs is limited by issues such as instability, binding with major blood proteins, and potential toxicity. Further research is necessary to optimize their use in medical applications [89]. Thus, while AgNPs have the potential to target granulomas, addressing their limited permeability and associated challenges is essential to utilize their therapeutic benefits.

### 4.3. Immunomodulation

The immunomodulatory effects of AgNPs have been extensively studied, including both anti-inflammatory and pro-inflammatory responses. AgNPs engage with diverse immune cells and pathways, impacting cytokine production, cell proliferation, and the activation of immune cells. AgNPs have significant immunomodulatory effects on macrophages, affecting their function in various ways. The size, surface coating, and concentration of AgNPs can determine whether they induce pro-inflammatory or anti-inflammatory responses [90]. For example, AgNPs have been found to decrease the secretion of pro-inflammatory cytokines in response to lipopolysaccharide (LPS) by inhibiting Toll-like receptor (TLR) signaling pathways, which are crucial for macrophage activation [91]. A study has shown that AgNPs reduce pro-inflammatory cytokines like IL1β, IL6, and TNFα, thus promoting wound healing. The study evaluated the wound-healing effect of AgNPs alone and in combination with low-level laser therapy. It was observed that AgNPs enhanced wound closure and reduced inflammation in albino mice [90]. In peripheral blood mononuclear cells (PBMCs), AgNPs did not induce significant cytotoxicity. Instead, they demonstrated a downregulation of pro-inflammatory genes, specifically IL1β and CCL3, in monocyte subsets, suggesting a potential for suppressing inflammatory responses [92]. AgNPs can hinder monocyte-to-macrophage differentiation by blocking autophagy and causing lysosomal dysfunction. This inhibits the expression of surface markers like CD11b and reduces the response to LPS stimulation [93]. The dimensions and surface chemistry of AgNPs exert a significant influence on their interaction with macrophages. Specifically, smaller particles and certain coatings have been observed to promote heightened macrophage uptake, consequently eliciting immunomodulatory effects. This includes the induction of the release of pro-inflammatory cytokines such as IL-2, IL-17A, and TNFα [94]. AgNPs can also interfere with NF-κB signaling pathways by suppressing the expression of key cytokines like IL-1β, which is crucial for host resistance to MTB [95]. The encapsulation of AgNPs within liposomes, known as Lipo-AgNP, has been shown to significantly enhance their cytotoxic effects on macrophages, while concurrently reducing the generation of reactive oxygen species (ROS). This phenomenon leads to apoptosis through a redox imbalance and increased DNA damage [96]. These various effects underscore the complex and context-specific nature of the immunomodulatory effects of AgNPs on macrophages, emphasizing the need for a careful consideration of their design and utilization in medical and consumer goods [97,98]. Sarkar et al. (2018) observed that exposure to AgNPs resulted in an upregulation of macrophage Hsp72 expression. This upregulation is postulated to be associated with the concurrent suppression of the NF-κB pathway, leading to diminished antimycobacterial activity in macrophages. This discovery is of particular significance in the clinical understanding of MTB complex (MTBC) infection and the polarization of macrophages [91]. An in vivo study on the toxicity of AgNPs in rats showed that high doses of AgNPs led to a pronounced suppression of natural killer cell (NK) activity [99]. In the presence of LPS, AgNPs led to heightened IL-1 production while concurrently diminishing the levels of IL-6, IL-10, and TNF in the spleen [100]. A study showed that neutrophils exposed to AgNPs (50 µg/mL) had decreased neutrophilic degranulation but the total phagocytic activity was increased [100]. The production of the inflammatory cytokine interleukin-6 and an improvement in phagocytic function in response to LPS stimulation were both stimulated by pre-exposing macrophages to AgNPs [101]. Additionally, when J774 A1 murine macrophages were exposed to AgNPs, IL-1, IL-6, and TNF-α gene expression increased earlier than when exposed to gold nanoparticles (AuNPs), signaling the inflammatory response [102]. In zebrafish models, AgNPs demonstrated a significant impact on the function and quantity of neutrophils and macrophages, suggesting a potential for innate immune toxicity. The natural compound pterostilbene exhibited the ability to alleviate these effects [103]. The introduction of gold nanoparticles as an alloy significantly improved AgNP biocompatibility [104]. AgNPs have been identified as activators of dendritic cells, thereby amplifying their capacity to combat intracellular infections. This activation is substantiated by the heightened expression of phenotypic markers and increased cytokine transcription [105]. AgNPs demonstrate potential immunomodulatory properties; however, their effects are subject to contextual variations influenced by particle size, surface functionalization, and biological environment. Further research is necessary to comprehend these interactions comprehensively and to refine their application in biomedical contexts.

### 4.4. Mycobacterial Resistance to AgNPs

The resistance to AgNPs in *Mycobacterium* species, including MTB, is influenced by various factors, such as genetic mutations, cell envelope characteristics, and nanoparticle properties. A comprehensive understanding of these mechanisms is crucial for the development of effective antimycobacterial strategies utilizing AgNPs. Resistance to AgNPs can emerge due to genetic mutations altering the interaction between bacterial cells and AgNPs. For instance, *M. smegmatis* mutants exhibited resistance to AgNPs following exposure, indicating that genetic modifications may confer resistance not only to silver, but also to specific antibiotics such as isoniazid [106]. The distinctive cell envelope of mycobacteria, which contains a lipid-rich outer membrane, significantly contributes to its resistance. This structure can hinder the penetration of AgNPs, thereby reducing their effectiveness. The existence of mycolic acids and specific transport proteins, such as porins, can impact the absorption and efficiency of nanoparticles [107]. The size and shape significantly influence the antimicrobial activity of AgNPs. Smaller nanoparticles, with a higher surface area-to-volume ratio, exhibit enhanced interaction with bacterial cells. Research has demonstrated that tetrahedral AgNPs, averaging 45 nm in size, effectively inhibit various strains of Mycobacterium, including multidrug-resistant ones [66,67]. The antimicrobial properties of AgNPs can be influenced by the method of synthesis. Chemically synthesized AgNPs have demonstrated higher efficacy compared to biologically synthesized ones, possibly due to differences in surface chemistry and stability [108]. Utilizing nanocarriers, such as mesoporous silica, to deliver AgNPs can mitigate toxicity and improve targeting to mycobacterial cells. This strategy has shown promise in reducing the minimum inhibitory concentration required for effective treatment [41].

**Figure 2:**
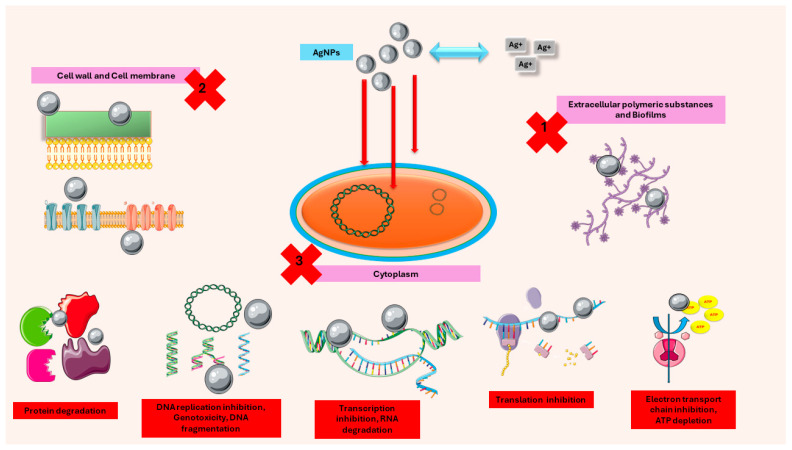
The antimicrobial activity of AgNPs is achieved through three primary mechanisms: 1. The accumulation and disruption of the extracellular polymers present in bacterial biofilms. 2. The adherence of AgNPs to the surface of bacterial cells, resulting in the disruption of microbial membranes, altered transmembrane transport, and leakage of cellular contents, ultimately leading to bacterial cell death. 3. The penetration of AgNPs into the microbial cytoplasm, where they can interact with organelles, causing protein degradation, disrupting metabolic pathways including the inhibition of DNA replication, DNA fragmentation, the inhibition of transcription, RNA degradation, translation inhibition, and the inhibition of the electron transport chain, causing ATP depletion. The figure was produced using Servier Medical Art.

Ag-resistance, which is typically seen in rapidly proliferating bacteria, has also been noted in *Mycobacteria*, including *M. smegmatis* [106], *M. avium*, *M. fortuitum*, and *M. mucogenicum*. *M. smegmatis* and other saprophytic bacteria were shown to be resistant to both AgNPs and AgNO_3_, and this was also demonstrable for other pathogenic bacteria. The Ag resistance mechanism may involve the removal and neutralization of silver ions, such as the active efflux of Ag^+^ (e.g., by P-type ATP/SilP), increased ability to reduce Ag^+^ to a neutral-oxidation state, which is often less bacteriotoxic, or membrane potential-dependent three-polypeptide cation/proton antiporter or multidrug resistance/MDR efflux pumps [109,110,111]. Recently, bacterial flagellum protein flagellin overexpression in Gram-negative bacteria caused particle aggregation at the surface of the bacteria and a decrease in the antibacterial action of AgNPs [112]. The complexity of the antibacterial mechanisms exhibited by AgNPs (as illustrated in Figure 2), compared to conventional antibiotics, presents a formidable challenge for bacteria to develop resistance to AgNPs. Furthermore, biofilm production of exopolysaccharides acts as a defense mechanism against AgNPs, enhancing bacterial survival [113]. Despite these resistance mechanisms, combining AgNPs with traditional antibiotics has shown promise in overcoming resistance. This is demonstrated by the enhanced efficacy of such combinations against multidrug-resistant strains of *E. coli* and *S. aureus* [114]. The development of functionalized AgNPs and the use of biological synthesis methods have also been investigated to enhance antimicrobial activity and reduce resistance [36,115]. AgNPs continue to demonstrate efficacy as an antimicrobial agent. However, the widespread utilization of these nanoparticles necessitates a meticulous assessment of the potential for resistance development, urging the implementation of tailored strategies to address this concern. 

Silver nanoparticles hold promise as therapeutic agents against mycobacterial infections; however, their clinical application is hindered by potential toxicity to human cells. Furthermore, the effectiveness of AgNPs may be compromised by various bacterial resistance mechanisms. The absence of standardized protocols for synthesis and characterization also presents challenges in comparing results across different studies. Additionally, the long-term stability and bioavailability of AgNPs in biological systems require further investigation to ensure their safe and effective use.

## 5. Conclusions

Combining AgNPs with traditional anti-TB therapies is a promising tactic, as they can work synergistically to enhance antimycobacterial action both extracellularly and intracellularly. AgNPS have demonstrated potent antimycobacterial activities, and when used in conjunction with conventional anti-TB drugs, they can overcome drug resistance mechanisms and improve treatment efficacy. The unique physiochemical properties of AgNPs, such as their small size, high surface area-to-volume ratio, and ability to penetrate mycobacterial cell walls and membranes, make them effective antimycobacterial agents. Inside the host cells, AgNPs can disrupt essential cellular processes, generate oxidative stress, and interfere with the intracellular survival mechanisms of mycobacteria. This dual mode of action, both extracellularly and intracellularly, is a significant advantage of combining AgNPs with traditional anti-TB therapies.

Despite the promising in vitro study results demonstrating the antimycobacterial potential of AgNPs, there is a notable lack of in vivo studies exploring their anti-TB applications. Translating these laboratory findings into animal models and, ultimately, clinical trials is crucial to evaluate the therapeutic efficacy and safety of AgNP-based treatments for TB.

The synthesis of novel nanoparticles with an increased local availability of these antimycobacterial nanoparticles at the site of mycobacterial infection can enhance the efficacy of the AgNPs. The unique shape, visceral distribution, and TB-induced lesions can influence the targeting and accumulation of nanoparticles within the affected tissues and cells. To be a suitable candidate for future tuberculosis therapy, a nanoparticle should possess a combination of desirable qualities: higher antimycobacterial activity depicted by low MICs, efficient intracellular delivery and availability within macrophages, low cytotoxicity, and increased local availability at the site of infection. By addressing these key design criteria, researchers can develop more effective and safer nanoparticle-based therapies for treating drug-resistant tuberculosis and other mycobacterial diseases.

While AgNPs offer a promising alternative to traditional antibiotics, their use is not without challenges. The potential for developing resistance, as seen with other antimicrobial agents, necessitates carefully considering dosing and delivery methods. Additionally, the environmental and health impacts of widespread AgNP use must be evaluated to ensure sustainable and safe application in medical and industrial settings. Further research into the mechanisms of resistance and the development of innovative delivery systems will be crucial in harnessing the full potential of AgNPs in combating mycobacterial infections.

To address the existing limitations of AgNPs in tuberculosis treatment, several potential enhancements can be considered. First, optimizing formulations to improve biocompatibility and targeted delivery could help reduce toxicity to human cells while maximizing efficacy against mycobacteria. Second, exploring combination therapies that incorporate AgNPs with established antituberculosis medications may enhance treatment outcomes and decrease the risk of bacterial resistance. Furthermore, enhancing the stability and bioavailability of AgNPs through advanced coating materials or encapsulation techniques can ensure more consistent therapeutic effects within biological systems. Establishing standardized protocols for the synthesis and characterization of AgNPs will facilitate comparison across studies and improve reproducibility. Finally, conducting well-designed clinical trials will yield vital data on the safety, efficacy, and pharmacokinetics of AgNPs, paving the way for their potential application in tuberculosis treatment.

## Figures and Tables

**Table 1:** Summary of in vitro studies evaluating the anti-mycobacterial activity of AgNPs.

Authors	Mycobacterial Species/Strains	Experimental Model	Dose	Shape of Nanoparticle	Size of Nanoparticle	Effect Observed
Avaliani et al., 2022 [36]	50 Fluoroquinolone-resistant *M. tuberculosis*	Bacteria	0.5 to 2 μg/mL	Spherical	20 nm	Bactericidal, 100% inhibition with a standard dose of Moxifloxacin combined with 10% AgNPs.
Kiria et al., 2022 [37]	Rifampicin-resistant *M. tuberculosis*	Bacteria	0.5 to 8 μg/mL	Spherical	20 nm	Growth inhibition.
Selim et al., 2018 [38]	*M. bovis*, *M. tuberculosis* H37Rv, MDR strains of *M. tuberculosis* and clinical isolates of *M. bovis* and *M. tuberculosis*	Bacteria	MICs of *M. bovis* (1 μg/mL), *M. tuberculosis* H37Rv (4 μg/mL), and MDR strains of *M. tuberculosis* (16 μg/mL). Clinical isolates of *M. bovis* and *M. tuberculosis* (MIC 4–32 μg/mL and 1–16 μg/mL)	Tetrahedral	50 nm	Growth inhibition by interaction with bacterial cell membranes, affecting cell viability by reacting with sulfur-containing amino acids and inhibiting enzymatic functions.
Patil et al., 2016 [39]	*M. tuberculosis*	Bacteria	MIC of 3.12 μg/mL	Spherical	21–42 nm, green synthesis using *Limonia acidissima* L. leaf extract	Growth inhibition.
Montalvo-Quirós et al., 2021 [41]	*M. tuberculosis* H37Rv	Bacteria	500 mg/mL	Not mentioned	Metallic silver nanoparticles core of AgMSNs, 77 nm, MSNs-AgBrNPs, hydrodynamic size of around 245.3 nm	Bacterial growth inhibition via affecting bacterial metabolic processes, DNA interaction, and cytoplasmic membrane permeability.
Song et al., 2006 [42]	*M. tuberculosis*	Bacteria	0.5–30 ppm	Not mentioned	5 nm	Bactericidal.
Singh et al., 2016 [43]	*M. tuberculosis* and *M. bovis* BCG.	Human cell lines THP-1, A549, and PANC-1	0.02 to 2.56 mg/mL	Not mentioned	10–120 nm	Growth inhibition.
Kote et al., 2016 [44]	*M. tuberculosis* (MTCC-300), *M. phlei* (MTCC-1723), *M. avium* (MTCC-1724), and *M. smegmatis* (MTCC-994).	Bacteria	1 to 10 μg/mL	Square-type nanocrystalline	Not mentioned	Bactericidal.
Raja et al., 2016 [45]	*M. smegmatis* and *M. tuberculosis*	Bacteria	1–5 μg/mL	Spherical	38–52 nm, green synthesis from *Catharanthus roseus*	Bactericidal.
Paarakh 2017 [46]	*M. tuberculosis* H37Rv	Bacteria	0.2–100 µg/mL	Spherical	100 nm, green synthesis from *Coriandrum sativum*	Growth inhibition.
Agarwal et al. 2013, [44]	*M. tuberculosis* H37Rv, 26 clinical isolates comprising drug-sensitive (DS), multidrug-resistant (MDR), extensive-drug-resistant (XDR), and Mycobacterium other than tuberculosis (MOTT) strains	Bacteria	7.8 to 25 µg/mL	Spherical	10–20 nm	Bactericidal via cell membrane disruption and immediate dissipation of proton motive force.
Daniel, et al. 2014, [48]	*M. smegmatis*	Bacteria	2 to 10 mg	Not mentioned	30–130 nm, green synthesis using Ipomea carnea	Bactericidal via disruption of bacterial cell membrane function.
Kote et al., 2014 [49]	*M. tuberculosis*, *M. smegmatis*, and *M. phlei*	Bacteria	100 µL to 500 µL	Not mentioned	7.23–13.73 nm, green synthesis using *Psidium guajava*	Growth inhibition.
Banu et al., 2013 [51]	Clinical isolates of *M. tuberculosis* (multidrug-resistant and extensive-drug-resistant) and Mycobacterium other than tuberculosis (MOTT)	Bacteria	6.25 to 12.5 μg/mL for *M. tuberculosis* and 0.2 to 100 μg/mL against resistant strains of *M. tuberculosis*	Spherical	3–20 nm, green synthesis using R. stolonifer	Bactericidal via interaction with sulfur-containing amino acids and phosphorus moieties in nucleic acids, inhibition of respiratory chain enzymes, and interference with membrane permeability.
Heidary et al. 2019, [52]	*Mycobacterium tuberculosis* (multidrug-resistant and extensively drug-resistant strains) and H37Rv	Human cell line THP-1	1 to 128 µg/mL	Spherical	2.6–5.4 nm	Bacteriostatic.
Ellis et al., 2018 [53]	*Mycobacterium tuberculosis* H37Ra	Human cell line THP1	60 mg/ mL	Not mentioned	20 nm	Bactericidal via lysis and increased membrane permeability, promoting the entry of rifampicin into the bacterial cytosol, ultimately enhancing the drug’s potency.
Jafari et al., 2017 [54]	*M. tuberculosis* H37Rv and *M. tuberculosis* ATCC 27294	Human cell line THP-1	0.663 ppm	Spherical	13 nm.	Growth inhibition.
Mohanty et al., 2013 [55]	*M. tuberculosis*, *M. avium*, *M. bovis*, *M. smegmatis*, and *M. marinum*,	Bacteria and Mouse cell line RAW264.7	0.1 to 0.5 ppm	Spherical	50 nm–100 nm, green synthesis using *Alstonia macrophylla* and *Trichoderma* sp.	Bactericidal.
Patel et al., 2024 [56]	*M. smegmatis* and *M. fortuitum* and *M. marinum*	Bacteria	100 μg/mL	Spherical	15–60 nm, green synthesis using *Clerodendrum serratum*	Growth inhibition.
Sivaraj et al., 2020 [57]	*M. tuberculosis* H37Rv and *M. smegmatis* MC2155	Bacteria	37 µg/mL	Spherical	9 nm–51 nm	Bactericidal via penetration of bacterial cell membranes, interference of ATP production, inhibition of DNA replication, and induction of oxidative stress.
Patel et al., 2018 [58]	*M. tuberculosis* H37Rv	Bacteria	12.5 mg/mL	Spherical	20 nm–56 nm, green synthesis using *Sesbania grandiflora*	Growth inhibition.
Aman et al., 2023 [59]	*M. smegmatis* and *M. tuberculosis*	Bacteria	5 mg/mL	Spherical	40 nm, green synthesis using Citrus pseudolimon	Growth inhibition.
Dhanislas et al., 2023 [60]	*M. smegmatis*	Bacteria	250 and 500 μg/mL	Not mentioned	20–149 nm, green synthesis using *Syzygium aromaticum*	Growth inhibition.
Jena et al., 2012 [61]	*M. smegmatis*	Mouse cell line RAW264.7	1 to 3 ppm	Not mentioned	Not mentioned, chitosan-stabilized silver nanoparticles	Bactericidal via disruption of bacterial cell membrane.
Abdel-Aziz et al., 2020 [62]	*M. tuberculosis*	Human cell lines A-549 and WI-38	1.95 µg/mL	Spherical	11 to 17.5 nm	Bactericidal via cell wall and cell membrane damage leading to cytoplasmic leakage and damage to the intracellular structures.
Seth et al., 2010 [63]	*M. tuberculosis* H37RV, clinical MTB strains, and *M. xenopi*	Bacteria	1.6–8 µg/mL	Not mentioned	6–45 nm	Bactericidal via disruption of the bacterial cell membrane.

**Table 2:** Summary of pre-clinical and clinical studies evaluating the anti-mycobacterial activity of AgNPs.

Authors	Mycobacterial Species/Strains	Experimental Model	Dose	Shape of Nanoparticle	Size of Nanoparticle	Effect Observed
Kalmantaeva et al., 2020 [71]	*M. tuberculosis* H37Rv	Mice	0.1 mg/kg administered via inhalation	Not mentioned	43.6 ± 10.7 nm	Growth inhibition in vitro, a twofold decrease in the colonization of the lungs and spleens by *M. tuberculosis* in infected mice
Chen et al., 2021 [40]	*M. tuberculosis* H37Ra, H37Rv, W6 (Beijing strain), KVGH376, KVGH264 (MDR strains), TCHL002, TCHL017 (XDR strains), CHCH005 (Beijing strain), and CHCH029 (East African-Indian strain)	Bacteria, human cell line THP-1, zebrafish, and mice	10, 50, 100 µg/mL, and 200 μg/mL ALG-AgNPs for 5 days in zebrafish, followed by 500 mg/kg doses by oral gavage or 250 mg/kg by intravenous injection	Spherical	50 nm	Bacterial growth inhibition, anti-mycobacterial potential in both zebrafish and mouse TB animal models
Zakharov et al., 2017 [70]	*M. tuberculosis* and multiple drug-resistant strains	Mice	2.5–50 µg/mL	Not mentioned	3 to 60 nm	Bactericidal
Uraskulova et al., 2017 [72]	Drug-resistant strains *M. tuberculosis*	Bacteria and clinical study on TB patients	3.3% solution	Not mentioned	A medical-grade polymer-stabilized silver cluster	Bactericidal, inhalation of the 3.3% argovit-C solution twice daily for 10 min over 2 months was more effective in treating laryngeal tuberculosis than standard anti-tuberculosis treatment as determined by sputum culture for MTB.

## Data Availability

No new data were created or analyzed in this study.
